# Special Issue “Molecular Advances in Cancer Genetics 3.0”

**DOI:** 10.3390/ijms25052717

**Published:** 2024-02-27

**Authors:** William Bruno, Paola Ghiorzo

**Affiliations:** 1 Genetics of Rare Cancers, IRCCS Ospedale Policlinico San Martino, Largo Rosanna Benzi 10, 16132 Genoa, Italy; paola.ghiorzo@unige.it; 2 Department of Internal Medicine and Medical Specialties (DIMI), University of Genoa, Viale Benedetto XV 6, 16132 Genoa, Italy

The third volume of this Special Issue focuses on new advances in cancer genetics studies and collates papers reporting on a variety of mechanisms of tumorigenesis, the need to explore them from multiple perspectives, and the difficulties in exploring them, as well as the challenge of integrating them into a unifying but still different model for each tumor type.

The collected studies illustrate the role of miRNAs in promoting tumor formation, provide descriptions of the pathways involved in the alteration of potential new tumor suppressor genes, and discuss the potential of new methods, such as cfDNA and ctDNA analysis, to improve patient monitoring and management, including therapeutic ones.

The search for and validation of alternative methods to classical surveillance and therapeutic markers has brought these assays, which have some now well-known advantages, such as their minimal invasiveness and the cost reductions associated with the next-generation sequencing approaches on which they are based, to the forefront.

On the other hand, each new method requires validation and results on large sets of cases to be compared [1], in addition to the need to refine the differentiation between cfDNA and ctDNA [2,3,4].

Furthermore, it cannot be ruled out that a single methodological approach will not identify all the information that may be retrieved and related to tumor progression, given the complexity of the mechanisms involved, including the tumoral microenvironment or immune response.

One example is the identification of a mechanism of MiR-199a-5p-mediated *SMARCA4* gene upregulation in Head and Neck Squamous Cell Carcinomas (HNSCCs), an event involved in cell invasion and metastasis [5]. HNSCCs are an excellent example of tumor heterogeneity under a generic label because of their associated, and often intertwined, risk factors and the different genomic alteration landscapes that characterize each subgroup [6,7,8], resulting from different underlying tumorigenesis processes.

As illustrated by Huang et al. [9], the pathways potentially involved are numerous, and therefore it is difficult to focus on the alteration of a single gene. Genetic profiling is also problematic in some cases due to the anatomical site of some malignancies. Hence, there is a need for additional disease monitoring tools.

However, because the relevant data are still preliminary, gaining clinical insights into ctDNA and its future trajectory is challenging. Questions can be raised about the limits of ctDNA, including the low sensitivity limit of the method and its reliability, which depends on the determination of tumor burden and metastases sites prior to defining useful biomarkers for disease tracking and recurrence prediction [10,11].

Interestingly, *SMARC4* is the same gene mentioned by Shaykevich et al. [12], as BRG1, whose role as a tumor promoter is illustrated. As described in the aforementioned paper, BRG1/SMARC4 has a role in chromatin remodeling as a catalytic subunit of the SWI/SNF complex, but it also has a role in autophagy and apoptosis. Furthermore, it seems to interact with the Ras/Raf/MAPK/ERK1/2 pathway. *BRG1*/*SMARC4* overexpression is found in different cancers, such as breast cancer, colorectal cancer, and prostate cancer, apparently as a result of an oncogene-like activity. Nevertheless, the prognosis is worse in Non-small Cell Lung Cancers with a suppressed expression of *BRG1*/*SMARCA4*, which, indeed, may act differently to other tumors [13,14].

It seems plausible to hypothesize that the two examples of the regulation of the same gene may intertwine and each represent a piece of a larger picture of a number of specific mechanisms which may differ for diverse types of neoplasia.

These scattered examples are also indicative of the fragmentation of knowledge and the degree of intricacy that researchers need to address and recognize so that the data generated by basic research can provide starting points for translational research, offering more and more links to be exploited for increasingly personalized medicine.

The challenge is to bring it all together and translate an ever-growing amount of knowledge about the extremely complex phenomenon of cancer progression into viable, validated, and effective clinical management models.
